# Bee Viruses: Routes of Infection in Hymenoptera

**DOI:** 10.3389/fmicb.2020.00943

**Published:** 2020-05-28

**Authors:** Orlando Yañez, Niels Piot, Anne Dalmon, Joachim R. de Miranda, Panuwan Chantawannakul, Delphine Panziera, Esmaeil Amiri, Guy Smagghe, Declan Schroeder, Nor Chejanovsky

**Affiliations:** ^1^Institute of Bee Health, Vetsuisse Faculty, University of Bern, Bern, Switzerland; ^2^Agroscope, Swiss Bee Research Centre, Bern, Switzerland; ^3^Laboratory of Agrozoology, Department of Plants and Crops, Faculty of Bioscience Engineering, Ghent University, Ghent, Belgium; ^4^INRAE, Unité de Recherche Abeilles et Environnement, Avignon, France; ^5^Department of Ecology, Swedish University of Agricultural Sciences, Uppsala, Sweden; ^6^Environmental Science Research Center, Faculty of Science, Chiang Mai University, Chiang Mai, Thailand; ^7^Department of Biology, Faculty of Science, Chiang Mai University, Chiang Mai, Thailand; ^8^General Zoology, Institute for Biology, Martin-Luther-University of Halle-Wittenberg, Halle (Saale), Germany; ^9^Halle-Jena-Leipzig, German Centre for Integrative Biodiversity Research (iDiv), Leipzig, Germany; ^10^Department of Biology, University of North Carolina at Greensboro, Greensboro, NC, United States; ^11^Department of Entomology and Plant Pathology, North Carolina State University, Raleigh, NC, United States; ^12^Department of Veterinary Population Medicine, College of Veterinary Medicine, University of Minnesota, Saint Paul, MN, United States; ^13^School of Biological Sciences, University of Reading, Reading, United Kingdom; ^14^Entomology Department, Institute of Plant Protection, The Volcani Center, Rishon LeZion, Israel

**Keywords:** bee, virus, transmission, *Apis*, non-*Apis*, natural infection, artificial infection

## Abstract

Numerous studies have recently reported on the discovery of bee viruses in different arthropod species and their possible transmission routes, vastly increasing our understanding of these viruses and their distribution. Here, we review the current literature on the recent advances in understanding the transmission of viruses, both on the presence of bee viruses in *Apis* and non-*Apis* bee species and on the discovery of previously unknown bee viruses. The natural transmission of bee viruses will be discussed among different bee species and other insects. Finally, the research potential of *in vivo* (host organisms) and *in vitro* (cell lines) serial passages of bee viruses is discussed, from the perspective of the host-virus landscape changes and potential transmission routes for emerging bee virus infections.

## Introduction

Viruses are omnipresent in practically all life forms, where they pose a potential threat to the health of the organism. This also applies for the viruses found in bees. Most of these viruses were originally discovered in honey bees, either through symptoms or diseases associated with infection. Some honey bee viruses can be propagated in, as well as isolated and purified from honey bee pupae. This enabled the initial characterization of the viruses and the development of diagnostic assays. With these early diagnostic assays, it was shown even then that these “honey bee viruses” could also be detected in other bee species and wasps, although no systematic host-range study has ever been conducted. However, recent studies found that those viruses are much more common and widespread than previously suspected, occurring in numerous other hymenopteran and non-hymenopteran arthropods. Furthermore, there are reports of spillover from and between hymenopteran taxa and non-hymenopteran arthropods. Knowing the different possible virus transmission routes and their potential host-range is key to understanding prevalence, epidemiology and virulence in different bee species. Numerous studies have recently reported the discovery and transmission routes of viruses in different arthropod species, including many bee species. The aim of this review is to provide an overview of the recent advances of the impact and transmission of viruses found in bees. We have divided this review into two main parts. The first part, ‘Natural infections,’ summarizes the current knowledge regarding natural infections and the transmission routes of viruses found in honey bees. The second part, ‘Artificial infections,’ provides an overview of the studies involving controlled experimental virus infections and transmission. The major modes and routes of virus transmission in bees are summarized in Box 1.

**Box 1. Virus transmission routes - Definitions for the present review.**

**Horizontal transmission** is the transmission of infectious agents among individuals of the same generation. Horizontal transmission of viruses in honey bees includes transmission to different bee developmental stages via oral and/or body contact. It includes indirect infections through contaminated food (food-borne transmission) and contact with feces; venereal transmission, where virus is transmitted from drones to queens during the nuptial flights or by artificial insemination, and vector-mediated transmission, where transmission is mediated by other organisms (vectors). This vector can either be a mechanical or biological vector. A mechanical vector is defined as an organism that transmits viruses without being infected itself, while in a biological vector the virus replicates in the vector organism before being transmitted.

**Vertical transmission** consists of the transmission of viruses to the next generation, which for honey bees is primarily from queens to their eggs. This transmission can be defined as either transovum or transovarial transmission, depending on whether viruses are transmitted on the egg surface or within the egg, respectively. A second form of vertical transmission is transspermal transmission, if the virus is present inside the sperm, which would be the drone equivalent of transovarial transmission. This has so far not been detected in honey bees.

**Transmission** is defined as the establishment of a new infection in a previously non-infected individual, after acquiring the virus inoculum directly from another infected individual, from a vector or indirectly from the environment. It excludes any passive acquisition or retention of virus inoculum that does not cause infection of the bodily tissues.

## Part 1: Natural Infections

Most of our understanding about natural bee virus infections came from studies with honey bees, particularly *Apis mellifera*. A big part of the recent developments in this field allowed for the expansion in knowledge of virus infections in other bee species. Most of these new developments involve bee species that can either be easily reared or are used for commercial pollination services, such as bumble bees (*Bombus* spp.), stingless bees (e.g., *Melipona* spp.), mason bees (e.g., *Osmia* spp.) and leafcutter bees (e.g., *Megachile* spp.). This section is therefore divided into five subsections: transmission of viruses in *Apis mellifera*, distribution of bee viruses in other *Apis* species, recently discovered bee viruses, transmission of bee viruses in non-*Apis* pollinators and transmission of viruses in non-bee insects.

### Transmission of Viruses in *Apis mellifera*

#### Horizontal Transmission

##### Oral-fecal

This route is arguably the most common route for bee virus transmission, both within honey bee colonies and between bee species (Figueroa et al., 2019). There are diverse and abundant evidence that supports this route of transmission for most viruses found in honey bees. Most bee viruses are shed in copious amounts into the feces, from where they are released into the environment and can be picked up by other bees, through floral networks (Figueroa et al., 2019). Feces are also sometimes shed within bee colonies, particularly when the weather (cold, rain, wind) prevents cleansing flights or when the bees suffer from diarrhea, usually due to indigestible compounds in honey or pollen, or for suffering from nosemosis, a disease caused by *Nosema* sp. Bees complete the transmission when attempting to remove these feces as part of their cleaning activities.

##### Trophallaxis

A foodborne transmission pathway involving trophallaxis (mouth-to-mouth sharing of food between colony members) was proposed to exist for Israeli acute paralysis virus (IAPV; Chen et al., 2014). Laboratory experiments showed that IAPV can indeed be acquired through trophallaxis (Amiri et al., 2019), but it is not known if this transmission also leads to infection.

##### Hypopharyngeal glands and larval food

The hypopharyngeal glands are paired tubular secretory organs in the frontal region of the worker bee head (Snodgrass, 1956). They secrete a proteinaceous substance, royal jelly, which is the principal component of larval food. Several viruses have been detected from worker bees hypopharyngeal glands and larval food indicating a potential virus transmission route. Acute bee paralysis virus (ABPV) is frequently detected in the hypopharyngeal glands of ABPV-symptomatic adults (Bailey and Milne, 1969). IAPV, a closely related virus of ABPV (de Miranda et al., 2010a), was also found at relatively high levels in hypopharyngeal glands (Chen et al., 2014). Deformed wing virus (DWV) has also been detected in the glandular secretions of nurse bees (Fievet et al., 2006) and in larval food (Yue and Genersch, 2005). Kashmir bee virus (KBV) and Sacbrood virus (SBV) have been detected in larval food (Shen et al., 2005a). The large amounts of Cloudy wing virus (CWV) in sealed brood suggest an oral transmission route, i.e., that nurse bees infected with CWV can transmit the virus to larva via the larval food (Carreck et al., 2010).

##### Other food sources

The detection of viruses in food sources (i.e., brood food, honey, pollen) also suggests an oral transmission route. KBV and SBV have been detected in honey, pollen, and royal jelly (Bailey and Fernando, 1972; Singh et al., 2010). *Apis mellifera* filamentous virus (AmFV) has been detected in honey and pollen (Gauthier et al., 2015). IAPV was found in pollen (Chen et al., 2014) while Black queen cell virus (BQCV), Lake Sinai virus (LSV), DWV, and SBV have been detected in pollen pellets (Singh et al., 2010; Ravoet et al., 2015a).

##### Detection in gut tissues and feces

Black queen cell virus was detected in queen gut tissues and feces, suggesting possible transmission through feces (Chen et al., 2006). However, BQCV seems to be partially dependent on *Nosema apis* for infection of adult bees by ingestion (Bailey et al., 1983a). Several other viruses have been detected in the feces of infected bees such as ABPV (Bailey and Gibbs, 1964), Chronic bee paralysis virus (CBPV; Bailey, 1965), DWV (Chen et al., 2006), KBV (Hung, 2000), IAPV (Chen et al., 2014), and CBPV contaminated feces have recently been proven to provoke CBPV infection and overt disease in naive bees placed in cages previously occupied with contaminated individuals (Ribière et al., 2007).

##### Topical or body contact

Chronic bee paralysis virus can be transmitted by topical application on newly denuded honey bee cuticula (Bailey et al., 1983b) and it is transmitted from contaminated bees to non-infected bees reared in the same cages (Amiri et al., 2014; Coulon et al., 2018). Similarly, IAPV can be transmitted by topical application to honey bee workers and subsequent physical contact between infected workers and queens that leads to highly infected queens, suggesting that IAPV can also spread through close bodily contact (Amiri et al., 2019).

##### Vector-mediated transmission

Virus transmission via another organism (a ‘vector’) is also considered a form of horizontal transmission, since it still concerns transmission between individuals of the same generation. The close interaction between honey bees with obligate parasites such as endo- and ectoparasitic mites create a scenario where such mites can act as either mechanical vectors (i.e., exclusively the physical transfer of the acquired virus to a new host) or even biological vectors (where the virus also replicates inside the vector) for viruses (Chantawannakul et al., 2006; Forsgren et al., 2009). Picorna-like virus particle aggregations were found in lysed cells from the body cavity of the tracheal mite, ***Acarapis woodi*** (Liu, 1991) which is an endoparasitic mite that lives inside the tracheae and air sacs of adult honey bees (Sammataro et al., 2000). Although the identity of the virus was not established, the particle sizes and shapes are one of the most common ones among insect viruses. The role of *A. woodi* as a vector for picorna-like viruses is still unclear, mostly because very little dedicated research has been conducted on the possible role of *A. woodi* as a vector of virus diseases, in part because in the past it has never been linked to any viral disease.

By contrast, there is abundant evidence for vectored virus transmission by the ectoparasitic mite ***Varroa destructor*** (Santillán-Galicia et al., 2010; Möckel et al., 2011; Martin et al., 2012; Gisder et al., 2018; Posada-Florez et al., 2019; Ryabov et al., 2019), which is the reason why this mite is currently the most damaging parasite of the honey bees (Rosenkranz et al., 2010). ABPV, KBV, and IAPV are part of a complex of related viruses (Chen and Siede, 2007; de Miranda et al., 2010b), sometimes referred to as the “AKI complex.” These viruses have been associated with honey bee colony losses, particularly when colonies are co-infected with *V. destructor* (Cox-Foster et al., 2007; de Miranda et al., 2010a; Dainat et al., 2012). As yet, there has not been any direct evidence of ABPV replication within varroa mites (Ball, 1983, 1985; de Miranda et al., 2010b). However, high levels of ABPV have been detected in individual varroa-parasitized bees, as well as in entire honey bee colonies, indicating that the mite functions as a mechanical vector of ABPV (Bakonyi et al., 2002; D’Alvise et al., 2019). Effective transmission by *V. destructor* mites to a new host occurs after 36 h of acquiring the virus. DWV is widely detected in colonies infested by *V. destructor*. The mites function as a mechanical vector of DWV as they can transmit DWV during feeding activities (Ball, 1989; Bowen-Walker et al., 1999; Nordström, 2003; Shen et al., 2005b). Moreover, *V. destructor* has been described as a biological vector of DWV since the virus multiplies inside the vector, which is linked to the subsequent appearance of overt DWV infections in emerging bees (Gisder et al., 2009). DWV is a quasispecies made up of a cloud of variants. These variants can be divided into three master variants, DWV-A, -B, and -C (Mordecai et al., 2016c). The latest evidence suggests that DWV-B replicates inside *V. destructor* (Ongus et al., 2004; Campbell et al., 2016; Posada-Florez et al., 2019). DWV-B was originally named Varroa destructor virus 1 that was shown to be virulent when injected in high-titers into pupae or adult bees in cage experiments (Ryabov et al., 2014, 2019; McMahon et al., 2016; Gisder et al., 2018) but had surprisingly protective features when dominant in a colony despite the presence of *V. destructor* (Mordecai et al., 2016a). Interestingly, a positive correlation of DWV-B with bees dying over the winter period in stationary colonies were observed (Natsopoulou et al., 2017), however, these colonies nonetheless survived the overwintering period and did not collapse the following spring. This was not the case when colonies with high-titers of DWV-A and possibly DWV-C resulted in unexpected colony losses over the same overwintering period (Kevill et al., 2017). DWV-A is proposed to be mechanically vectored by *V. destructor* as it could only be transferred in lab-based experiments in a non-propagative manner (Posada-Florez et al., 2019).

Israeli acute paralysis virus is also widely detected in colonies infested by *V. destructor* mites. Evidence shows that *V. destructor* mites serve as an effective mechanical and biological vector of IAPV (Di Prisco et al., 2011). The detection of KBV in *V. destructor* mites and their salivary secretions (Hung and Shimanuki, 1999; Hung, 2000; Shen et al., 2005b) suggests that the parasite may act as a vector of KBV. CBPV was also detected in *V. destructor*, and the mite was involved in CBPV infection within the hive (Celle et al., 2008). *V. destructor* was also proven to acquire Slow bee paralysis virus (SBPV) by feeding on infected pupae and subsequently transmitting the virus to new parasitized pupae (Santillán-Galicia et al., 2010). *V. destructor* was proposed to be a biological vector for Apis rhabdovirus-1/Bee rhabdovirus-1 (ARV-1/BRV-1). Although replication of intermediate forms for these viruses were detected in varroa mites (Levin et al., 2017), the fact that much of the *V. destructor* gut contents, including nucleic acids and possible virus replication intermediate forms, are derived from their bee hosts (Cornman, 2017; Posada-Florez et al., 2019), means that such evidence is not necessarily conclusive for biological vector status of the mite (see also de Miranda et al., 2015). Nevertheless, Remnant et al. (2017) proposed that *V. destructor* is a genuine host for ARV-1 and -2 because the ssRNA (small RNA) profile of these viruses found in mites were different from those found in honey bees. *V. destructor*-mediated transmission of LSV is also suspected, as the virus is readily detected in the mite, although no causal association has been shown thus far (Daughenbaugh et al., 2015; Ravoet et al., 2015a). Bee Macula-like virus (BeeMLV) is also strongly correlated with the presence of *V. destructor*. It was shown to replicate in bees, and thus accumulate replication intermediates in mites (de Miranda et al., 2015). More conclusive evidence is needed to elucidate the precise role of mites in the transmission of this virus (de Miranda et al., 2015). Similarly, there is no conclusive evidence that SBV is directly transmitted by *V. destructor*, although it is frequently associated with varroa-related damage (Dubois et al., 2020), host-virus molecular interactions (Di Prisco et al., 2016; Ryabov et al., 2016; Remnant et al., 2019) and tolerance (Thaduri et al., 2018) as well as in *V. destructor* behavior (Giuffre et al., 2019). For BQCV, there is no evidence that it replicates in or is transmitted by *V. destructor*.

As in the case of the *V. destructor – Apis mellifera* relationship, Tropilaelaps mites are also ectoparasitic mites that are native to Asia and naturally parasitise *Apis dorsata*. Two species of Tropilaelaps mites (*Tropilaelaps mercedesae* and *T. clareae*) are also able to parasitise *Apis mellifera*. Compared with *V. destructor*, ***T. mercedesae*** is much more dependent on the continuous availability of honey bee brood for feeding and reproduction (Chantawannakul et al., 2016). *T. mercedesae* was also shown to transmit DWV in honey bees (Dainat et al., 2009; Forsgren et al., 2009; Khongphinitbunjong et al., 2016; Wu et al., 2017) and was associated with clinical DWV symptoms, such as reduced longevity, reduced weight at emergence and crippled wings (Khongphinitbunjong et al., 2016). ABPV, another common honey bee virus, has also been detected in *T. mercedesae*, with phylogenetic analyses implying that ABPV might have moved from *T. mercedesae* to *A. mellifera* (Chanpanitkitchote et al., 2018).

***Aethina tumida***, the small hive beetle (SHB), is a scavenger of honey bee colonies, whose larvae feed on honey, pollen and detritus. It has been shown that SHB can acquire DWV by feeding on DWV-infected brood and bees, by topical contact with DWV-contaminated wax and by exploiting trophallaxis between bees. The occurrence of high DWV titers in SHB suggests that it could be a true host, and possible biological reservoir, for DWV. Since SHB consumes bees, the detection of DWV replication intermediates (negative-strand RNA) is by itself not conclusive evidence for biological-vector status, similar as for varroa, but supports the evidence of quantitative increase in DWV titers (Eyer et al., 2009).

##### Venereal transmission

Venereal transmission is also considered a form of horizontal transmission, since it involves individuals from the same generation. Because of the challenges in experimentally controlling the natural mating process of honey bee queens and drones, much of the evidence for venereal infection is based on experiments with artificial insemination (Yue et al., 2006; de Miranda and Fries, 2008) the detection of viral particles in the reproductive organs, tissues and secretions of drones and queens (e.g., endophallus, semen, ovaries, spermatheca). That is the case for the detection of ABPV (Yue et al., 2006; Prodělalová et al., 2019), BQCV (Prodělalová et al., 2019), SBV (Prodělalová et al., 2019), AmFV (Gauthier et al., 2015; Prodělalová et al., 2019), IAPV (Chen et al., 2014), and DWV (Fievet et al., 2006; Yue et al., 2006; de Miranda and Fries, 2008; Yañez et al., 2012a; Prodělalová et al., 2019) in semen, which first identified the potential for sexual transmission. Similarly, the occurrence of viruses in the spermatheca of mated queens such as DWV (Chen et al., 2006; de Miranda and Fries, 2008; Francis et al., 2013) and IAPV (Chen et al., 2014) suggests the potential for virus found in sperm to cause infection in the queen tissues; confirming the possibility for sexual transmission through artificial insemination (Yue et al., 2007; de Miranda and Fries, 2008). However, the question whether venereal transmission also occurs naturally remains unresolved. The detection of high titers of DWV in the endophalluses of drones sampled from drone congregation areas (Yañez et al., 2012a), where they can potentially mate with queens during nuptial flights, as well as in the mating signs collected from returning queens (Amiri et al., 2016), showed that there were no functional consequences of high DWV loads in reproductive drones for natural venereal virus transmission. Furthermore, high viral titers were detected in some of the endophallic remains that were left inside mating organs of returning mated queens by the last mating drone (Amiri et al., 2016), again showing that such high-titers were no hinder to successful mating. Similarly, high DWV titers were found in the spermathecae and in the sperm contained in these for several of the tested queens (Amiri et al., 2016). These results provide evidence that DWV can be transmitted through both artificial and natural mating, confirming the earlier indirect evidence from artificial insemination (Chen et al., 2006; Yue et al., 2006, 2007; de Miranda and Fries, 2008).

#### Vertical Transmission

For honey bees, vertical transmission involves the transfer of viruses from queens or drones (either directly through sperm or indirectly through prior venereal infection of the queen) to their offspring. Several studies reported detection correlated distribution of viruses in queens and their eggs, which implies vertical transmission. For instance, BQCV and DWV were detected in all analyzed queens (*N* = 10) and in all pools of 50 eggs from these queens (Chen et al., 2006). Other indirect evidence relevant for vertical transmission is the presence of viruses in the queen’s reproductive organs. BQCV was detected in 70% of queen ovaries while DWV was detected in the ovaries of all analyzed queens (Chen et al., 2006). Francis et al. (2013) also showed the high prevalence of DWV in queen’s ovaries with 80% (*N* = 86) testing DWV positive. Moreover, Fievet et al. (2006) showed that the ovaries were the organs with the highest DWV titers in their tested queens. de Miranda and Fries (2008) traced DWV through the entire venereal-vertical infection process, from artificial insemination through infection of the spermatheca and queen’s ovaries to the resulting offspring, while Amiri et al. (2016) showed that ovaries can be infected with DWV after natural mating with DWV-positive drones and the virus can afterward be passed on to the eggs laid by these queens (Amiri et al., 2018). Not all the offspring from DWV-infected queens are DWV positive, nor do all DWV-infected queens also transmit vertically (Yue et al., 2007; de Miranda and Fries, 2008), so barriers to vertical transmission do exist, but in general terms the accumulated evidence indicates that DWV uses vertical transmission as a natural route for dissemination.

Regarding other viruses, the detection of CBPV (Chen et al., 2005, 2006; Blanchard et al., 2007; Ravoet et al., 2015b), IAPV (Chen et al., 2014), KBV (Chen et al., 2005; Shen et al., 2005a), SBV (Chen et al., 2005, 2006; Ravoet et al., 2015b), ABPV (Ravoet et al., 2015b), LSV (Ravoet et al., 2015b), Aphid lethal paralysis virus (ALPV, Ravoet et al., 2015b), and AmFV (Gauthier et al., 2015) in queens, their ovaries or in eggs, implies potential vertical transmission for these viruses as well. Additionally, the detection of BQCV, DWV, CBPV, KBV, and SBV in surface-sterilized eggs (Chen et al., 2006), strongly suggest transovarial transmission of these viruses, a pathway that involves the acquisition of the virus during oogenesis (however, see Amiri et al., 2018).

No information is available for vertical transmission of some less studied viruses, such as CWV, LSV, Moku virus (MV), BeeMLV, and/or ARV-1/BRV-1.

The present knowledge about which bee viruses are transmitted through various transmission routes is summarized in Table 1.

**TABLE 1:** Routes of infection of viruses associated with honey bees. Overview of their horizontal and vertical transmission routes.

Virus	Transmission					
	Horizontal					Vertical
	Oral*	Fecal	Body Contact	Venereal	Vector-mediated	Queen to eggs
IAPV	+	+	+	Ve.S.	+ (Vd)	+
ABPV	+	+	BC.S	Ve.S.	Vd.S., Tm.S.	+
KBV	+	+	BC.S	–	+ (Vd)	+
BQCV	+	+	?	Ve.S.	–	+
DWV	+	+	–	+	+ (Vd, Tm, At.S.)	+
SBV	+	–	–	Ve.S.	–	+
SBPV	+	?	?	?	+ (Vd)	?
CWV	O.S.	?	?	?	–	?
CBPV	+	+	+	–	Vd.S	+
LSV	+	?	?	–	Vd.S.	+
BeeMLV	?	?	?	?	Vd.S.	?
AmFV	+	?	?	Ve.S.	–	+
ALPV	?	?	?	?	?	+
ARV-1/BRV-1	?	?	?	?	Vd.S.	?
ARV-2/BRV-2	?	?	?	?	Vd.S.	?
ABV-1	?	?	?	?	?	?
ABV-2	?	?	?	?	?	?
ArkBV	?	?	?	?	?	?
BerkBPV	?	?	?	?	?	?
BSRV	?	?	?	–	?	?
C/TSBV	+	?	?	?	?	?
BVX	+	?	?	?	?	?
BVY	+	?	?	?	?	?
VTLV	?	?	?	?	Vd.S.	?
AIV	?	?	?	?	?	?
MV	?	?	?	?	?	?
VDV-2	?	?	?	?	?	?
VDV-3	?	?	?	?	?	?
VOV-1	?	?	?	?	?	?
AFV	?	?	?	?	?	?
ANV	?	?	?	?	?	?
ADV	?	?	?	?	?	?

ABPV, Acute bee paralysis virus; ABV-1, Apis bunyavirus-1; ABV-2, Apis bunyavirus-2; AmFV, Apis mellifera filamentous virus; ADV, Apis dicistrovirus; AIV, Apis iridescent virus (Bailey et al., 1979); ArkBV and BerkBPV, Arkansas bee virus and Berkeley bee virus (Bailey and Woods, 1974); AFV, Apis mellifera flavivirus; ANV, Apis Nora virus; ARV-1/BRV-1, Apis/Bee rhabdovirus-1; ARV-2/BRV-2, Apis/Bee rhabdovirus-2; BeeMLV, Bee Macula-like virus; BVX and BVY, Bee virus X and Y (Bailey et al., 1983a); BSRV, Big Sioux river virus (Runckel et al., 2011); BQCV, Black queen cell virus; IAPV, Israeli acute paralysis virus; C/TSBV, Chinese/Thai sacbrood virus; CWV, Cloudy wing virus; CBPV, Chronic bee paralysis virus; DWV, Deformed wing virus; KBV, Kashmir bee virus; LSV, Lake Sinai virus; MV, Moku virus; SBV, Sacbrood virus; SBPV, Slow bee paralysis virus; VDV-2 and VDV-3, Varroa destructor virus-2 and -3 (Levin et al., 2016); VOV-1, Varroa orthomyxovirus-1 (Levin et al., 2019); VTLV, Varroa Tymo-like virus (de Miranda et al., 2015). *, including trophallaxis, gut content and contaminated food; +, transmission confirmed; –, non-demonstrated transmission; ?, unknown; O.S., suggested oral transmission by presence in sealed brood; BC.S, suggested transmission by body contact; Ve.S., suggested venereal transmission by presence in semen and/or spermatheca; Vd, transmission confirmed by *Varroa destructor*; Vd.S., suggested vector-mediated transmission by *Varroa destructor*; Tm, transmission confirmed by *Tropilaelaps mercedesae*; Tm.S., suggested vector-mediated transmission by *Tropilaelaps mercedesae*; At.S., suggested vector-mediated transmission by *Aethina tumida.*

### Distribution of Bee Viruses in Other *Apis* Species

Bee viruses are capable of infecting multiple host species and horizontal transmission seems to play a crucial role in the global viral distribution patterns in different species of social honey bees (*Apis* spp.), which share ecological habitats and geographic ranges, particularly in south-east Asia. The most prevalent viruses in *Apis* species are DWV and BQCV, which for historical reasons were first described in the European honey bee, *A. mellifera* (Chen and Siede, 2007; Zhang et al., 2012), but have since also been detected in other honey bee species. DWV has been detected in four honey bee species (*A. mellifera*, *A. dorsata, A. florea* and *A. cerana*) (Chantawannakul et al., 2006; Berényi et al., 2007; Sanpa and Chantawannakul, 2009; Kojima et al., 2011; Ai et al., 2012b; Li et al., 2012; Forsgren et al., 2015; Yañez et al., 2016). Nevertheless, DWV occurs less frequently in wild honey bees (i.e., *A. florea* and *A. dorsata*) than BQCV (Zhang et al., 2012; Mookhploy et al., 2015). BQCV and the AKI viruses are also multi-host pathogens that can infect all honey bee species, as well as numerous non-*Apis* species. Since BQCV can be transmitted via contaminated food sources such as honey and pollen, this may be a route of transmission for honey bees residing in close proximity by sharing food sources.

The presence of KBV in *A. cerana* was first shown in bees from India (Bailey and Woods, 1977) and later also in South Korea (Choe et al., 2012). IAPV has also been detected in *A. cerana* (Kojima et al., 2011; Ai et al., 2012b; Yañez et al., 2016).

Sacbrood virus has been detected in several *Apis* species (Allen and Ball, 1996). It is especially prominent and damaging in *A. cerana* (Shah and Shah, 1988; Grabensteiner et al., 2001; Sanpa and Chantawannakul, 2009; Yoo and Yoon, 2009; Choi et al., 2010; Kojima et al., 2011; Ai et al., 2012a; Forsgren et al., 2015; Yañez et al., 2016). Thai sacbrood virus (TSBV, also known as Chinese sacbrood virus) was the first virus discovered in *A. cerana* from Thailand in 1976 (Bailey and Collins, 1982). TSBV also caused the death of more than 90% of domesticated *A. cerana* populations in Kashmir (Abrol and Bhat, 1990), and was found in *A. dorsata* and *A. florea* in India (Allen and Ball, 1996).

Multiple viral infections in individual bees or whole colonies have been reported in both managed and feral colonies of *Apis* species. This emphasizes the importance of virus–virus and bee–virus interactions (Takahashi et al., 2007; Choi et al., 2008; Sanpa and Chantawannakul, 2009; Ai et al., 2012a; Choe et al., 2012; Li et al., 2012; Ra et al., 2012; Reddy et al., 2013; Forsgren et al., 2015; Mookhploy et al., 2015).

During bee virus surveys in Asia, where both *A. cerana* and *A. mellifera* are cultivated, phylogenetic analysis of the capsid-protein gene of BQCV isolates from Thailand, China, South Korea, and Japan revealed a strong geographic clustering within Asia, distinct from South African and European isolates. No clustering were, however, observed according to the *Apis* host species that the isolates came from Mookhploy et al. (2015). BQCV isolated from Korea and Japan also showed similar levels of regional genetic variation, with high levels of similarity between isolates from the same country or continent (Kojima et al., 2011; Noh et al., 2013). That could be due to local transmission of viruses or spillover of BQCV from managed *A. mellifera* colonies to wild bees, as was the case for DWV spillover to local bumble bees (Fürst et al., 2014; Tehel et al., 2016). Another interesting point is that there appears to be no host-specific genetic adaptation by the virus when it is transmitted between bee species within the same geographic region (Fürst et al., 2014). It has been also suggested that in a two-host system, parasites may either evolve to be generalists, showing low levels of virulence, or specialists, displaying high virulence to each host species (Regoes et al., 2000). These studies highlight the complexity of bee virus disease ecology and transmission between their *Apis* and non-*Apis* hosts.

### New, Recently Discovered Bee Viruses

Many new bee viruses have been discovered recently through high throughput sequencing (HTS) technologies (reviewed in Beaurepaire et al., 2020). Most of these have only been characterized taxonomically, through phylogenetic analyses of their genomic sequences, without any information about their biological properties, including, crucially, whether the bee they were found in is actually a true host or not. This includes recently described viruses such as Moku virus (Mordecai et al., 2016b); Apis Nora virus (ANV), Apis bunya virus-1,2 (ABV-1, ABV-2), Apis dicistrovirus (ADV) and Apis flavivirus (AFV) (Remnant et al., 2017), AmFV (Gauthier et al., 2015), Apis rhabdovirus-1,2 (ARV-1,-2)/Bee rhabdovirus-1,2 (BRV-1,-2) (Levin et al., 2017; Remnant et al., 2017), as well as new viruses from the families *Iflaviridae*, *Tymoviridae*, *Nudiviridae*, and *Parvoviridae* (Schoonvaere et al., 2018) and new Tymo-, Seco-, Partiti-, Noda-, Dicistro-, Circo-, Nege-, Sobemo-, and Toti-like viruses (Galbraith et al., 2018; Schoonvaere et al., 2018); and the many picorna-like viruses identified in Australian honey bees (Roberts et al., 2018). Investigating the biological properties of these genetically characterized viruses is the next logical challenge, since just their geographic origin or distribution is not sufficient to infer the putative danger these new viruses represent for honey bees.

### Transmission of Bee Viruses in Non-*Apis* Pollinators

Most viruses that were first described in *Apis* sp. have also been detected in other bee species. Below we will give a short overview of the viruses detected in non-*Apis* species (for a more extensive review, we refer to Ravoet et al., 2014; Tehel et al., 2016; Gisder and Genersch, 2017).

Bumble bees are by far the most investigated non-*Apis* species with regard to viral infections and the presence of other pathogens (McMahon et al., 2015). Members of the DWV masters variants have been found in several *Bombus* sp. across different continents (Gisder and Genersch, 2017). Active replication of DWV in *Bombus* sp. has been confirmed in several Bombus species (Levitt et al., 2013; Fürst et al., 2014). Similarly for ABPV, IAPV and KBV infection in *B. terrestris* (Meeus et al., 2014), indicating that *Bombus* spp. are within the true host range of these viruses. Different viruses have different prevalences and titers in different bumble bee species (McMahon et al., 2015), which may reflect distinct susceptibilities, tolerances or transmission networks (Tehel et al., 2016; Figueroa et al., 2019). DWV infections have also been found in species of the genus *Augochlora*, *Ceratina*, *Xylocopa*, *Andrena*, *Heriades*, *Osmia*, *Melipona*, and *Scaptotrigona* (Singh et al., 2010; Guzman-Novoa et al., 2015; Tehel et al., 2016) and species belonging to Halictidae family (Evison et al., 2012; Levitt et al., 2013). Active DWV replication has been detected in *Osmia cornuta*. DWV titers in *M. subnitida* are similar to those found in honey bees. Both results suggest that DWV is capable of infecting and multiplying in at least *Osmia* spp. and *Melipona* spp. (Mazzei et al., 2014; de Souza et al., 2019). BQCV has been detected in several *Bombus* species as well as in *Melipona* and solitary bees of the genera *Xylocopa*, *Osmia*, *Andrena* and *Heriades* (Singh et al., 2010; Ravoet et al., 2014). Active replication of the virus has only been reported for *Bombus* spp. (Peng et al., 2011). The AKI virus complex has been detected in the genera *Bombus*, *Augochlora*, *Andrena*, *Heriades, Xylocopa* and *Melipona*, but active replication has only been shown in *Bombus* spp. (Singh et al., 2010; Levitt et al., 2013; Niu et al., 2016; Tehel et al., 2016; Alvarez et al., 2018) and bees from the Halictidae family (Levitt et al., 2013). So far infections with CBPV and SBPV have only been identified in *Bombus* sp., where active virus replication was verified only for SBPV (Niu et al., 2016).

Sacbrood virus was detected in *Bombus*, *Andrena, Ceratina*, and *Xylocopa* species (Singh et al., 2010; Levitt et al., 2013) and the Halictidae family (Levitt et al., 2013). Viruses of the LSV complex have been identified in *Bombus*, *Osmia*, and *Andrena* species, but virus replication has only been reported for *Bombus* sp. and *Osmia* sp. The species *Halictus scabiosae*, *Halictus sexcinctus*, and *Halictus simplex* were screened for viruses by Bigot et al. (2017), where the *Halictus scabiosae* Adlikon virus (HsAV) was described and identified as a virus closely related to LSV. ARV-1/BRV-1 was found in *Bombus* sp. (Levin et al., 2017). As apparent from this short overview, most of these viruses are detected in many different genera of bees. Therefore, it would be difficult to pinpoint the true host range of most viruses and our current knowledge is far from a clear understanding of the presence and replication of viruses in non-*Apis* and non-*Bombus* bee species. It is impossible to tell which bee species is the primary host, or even whether this question is relevant in the complicated context of bee virus transmission. Unraveling the directionality of virus transmission is therefore difficult from the current knowledge, based largely on natural surveys, but may benefit from systematic experimental approaches (e.g., Fürst et al., 2014; Meeus et al., 2014; Figueroa et al., 2019). That said, we will now break down the transmission routes into intra-species and inter-species transmission for non-*Apis* bee species.

#### Inter-Species Virus Transmission

**FIGURE 1:**
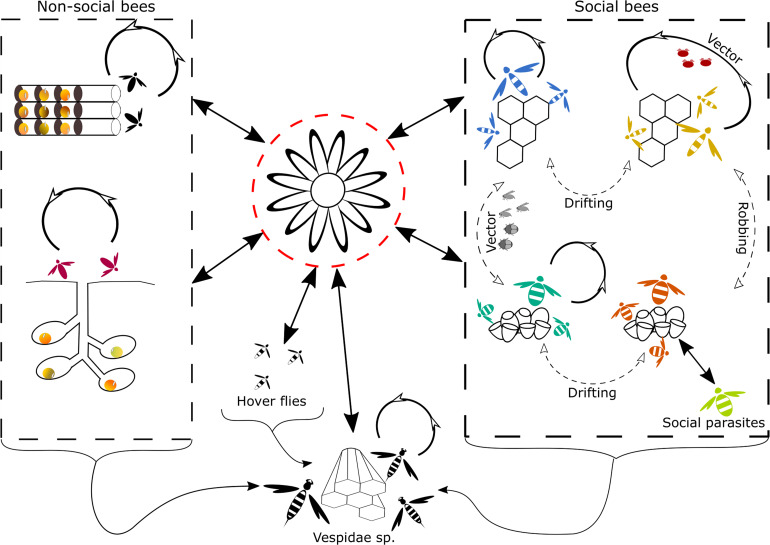
Graphical representation of the natural routes of inter- and intra-species transmission of viruses found in Hymenoptera. Transmission routes that are more likely to occur are represented by solid arrows, transmission routes that are less likely or less frequently observed are represented by dotted arrows. The most likely route of inter-species transmission is through the use of shared flowers (Singh et al., 2010; Alger et al., 2019), depicted in the middle surrounded by a red dotted line. Inter- and intra-species transmission in non-social bees, depicted on the left side can occur through contact at nest aggregations or the reuse of virus-contaminated old nest cavities (Krunicì and Stanisavljevicì, 2006). In social bees, depicted on the right side, the main intra-species transmission route is likely, via intense contact in the nest. Social behavior such as trophallaxis and body contact could mediate transmission (Amiri et al., 2014, 2019; Chen et al., 2014; Coulon et al., 2018 and others, see main text). Further intra-species transmission can also occur via vectors, for honey bees this is a very important transmission route for several viruses, mediated by *V. destructor* (Santillán-Galicia et al., 2010; Möckel et al., 2011; Gisder et al., 2018; Posada-Florez et al., 2019; Ryabov et al., 2019 and others, see main text). Intra-and inter-species transmission via vectors, other than *V. destructor* [e.g., the small hive beetle, phorid flies (Eyer et al., 2009; Core et al., 2012; Menail et al., 2016)] are less frequent. Robbing and drifting are also two potential routes of inter- and intra-species transmission, respectively, described in social bees. Yet their role in virus transmission is likely to be minor compared to other transmission routes. Lastly, social parasitism creates a high contact between individuals from different species and hence is likely to facilitate virus transmission, in social bees. Inter-species transmission in *Vespidae* sp. can occur through feeding on infected Hymenoptera, or other insects that are infected with a virus or contain the virus on their exterior (Loope et al., 2019). Another potential transmission route for wasps is the use of shared contaminated flowers (Mordecai et al., 2016b). Intra-species transmission in social *Vespidae* sp. is also likely to occur via intense contact within the nest.

Natural inter-species virus transmission can occur via several different routes (Figure 1). Oral-fecal transmission is most likely the main route of inter-species virus transmission. Several viruses such as CBPV, KBV, DWV, BQCV, and IAPV have been identified in the feces of honey bees (Hung, 2000; Chen et al., 2006; Ribière et al., 2007; Chen et al., 2014). Although no study to date has reported the presence of viruses in the feces of non-*Apis* species, one could expect that virus particles are shed via the feces, similar to how this happens in honey bees. Several studies have directly identified the role of shared flowers in the transmission of a number of bee pathogens, including viruses (Singh et al., 2010; Graystock et al., 2015; Adler et al., 2018; Alger et al., 2019). Therefore, infected bees visiting flowers can contaminate the flower surface, nectar and pollen with virus particles they shed via their feces. Studies have identified the potential of indirect virus transmission via shared flowers (Singh et al., 2010; Bodden et al., 2019).

Analysis of the corbicular pollen from foraging honey bees showed the presence of SBV, BQCV and DWV in the pollen. DWV was also found in pollen collected by non-*Apis* bees. As infected pollen could be found on non-infected bees and *vice versa* (Singh et al., 2010), one can conclude that corbicular pollen is not primarily contaminated by the bee itself, e.g., through salivary excretions, but rather by previous visits from infected bees (Singh et al., 2010; Figueroa et al., 2019). The viability of the DWV present on the pollen has been demonstrated by injection into honey bees and *Osmia* spp. (Mazzei et al., 2014), although this does not of course mean a similar viability for oral infection. Oral transmission requires many orders of magnitude greater quantities of virus (de Miranda et al., 2013). DWV is naturally notoriously unstable outside the cell (de Miranda et al., 2013), and other pathogens are also known to lose viability rapidly on flower petals (Figueroa et al., 2019).

Apart from shared flowers, which is most likely the main route of inter-species transmission, there are other interactions between bee species that could promote the transmission of viruses between bee species. Robbing is one such interaction, where a bee steals stored resources from another nest, whether from the same or from a different species. Robbing is a well-known phenomenon between honey bee colonies and occurs primarily during a dearth of floral resources (Kuszewska and Woyciechowski, 2014). Robbing of honey bee colonies by bumble bees (Genersch et al., 2006) and wasps has also been documented, but is more individual rather than systematic and usually toward the end of summer when both wasp and bumble bee colonies are in natural decline. Viruses can certainly be detected in honey (Milićević et al., 2018), so that robbing honey does expose robbing bees and wasps to potential infection (Genersch et al., 2006), but direct evidence for virus transmission through honey is so far absent, for honey bees or bumble bees.

Some social and solitary bees suffer from brood parasitism. This is when a different, parasitic bee species invades the nest of its host species and uses the host’s resources to its own reproductive benefit (Lhomme and Hines, 2018). Such intrusion also involves exposure to potentially infectious viruses present in the host bees and brood or on the nest structures, enabling inter-species transmission from the nest of the host to the brood parasites.

Although rare, there are reports of bumble bee species reusing old nests (Taylor and Cameron, 2003). As viral particles can build up in the nest during the development, reusing an old nest will expose the new colony to these viruses. However, viral particles present in the old nest are exposed to environmental conditions (e.g., humidity and heat) and can deteriorate, losing their infectivity. The environmental stability of viruses found in honey bees seems to be highly dependent on the virus, where some viruses deteriorate faster compared to others (Chen et al., 2007; Dainat et al., 2011; Forsgren et al., 2017). Forsgren et al. (2017) showed that BQCV remains detectable up to 4 days in dead bees stored at 4°C, whereas DWV degrades faster. One side note on these studies is that they do not assess the infectability of the virus. Because the environmental stability of the virus and the loss of its infectability likely differ between viruses present in a dead host and viruses that are outside their host, yet further research is still needed here.

The reuse of nesting sites is more common in solitary bees, which often re-occupy nesting sites used in previous years (Krunicì and Stanisavljevicì, 2006). As for bumble bees, old nest sites of solitary bees (i.e., hollow cavities in sand, stone, wood, or straw) can contain virus particles that were present on the pollen brought in by solitary bees the previous year. If these viruses are still contagious they could infect the adult female or the larvae of the next generation.

#### Intra-Species Transmission

The intra-specific transmission of viruses in non-*Apis* bees should also be highlighted. The very different lifestyles and social structures of non-*Apis* bee species will greatly affect how effectively, different bee viruses are transmitted.

Eusocial non-*Apis* species belong to the tribes *Meliponini* (stingless bees) and *Bombini* (bumble bees). Both tribes have a social structure similar to honey bees, with a single reproductive queen and non-reproductive worker bees attending the brood and foraging. A social nest structure creates a high contact between nest mates which facilitates virus transmission. Bees of the family *Halictidae* are widely distributed. This family displays a plasticity of social behaviors ranging from solitary to communal, semi-social and primitively eusocial (Danforth, 2002), allowing for different levels of horizontal transmission. Strictly solitary species on the other hand have very little contact with other individuals from the same species, apart from mating. Intra-species transmission here is most likely via the use of shared flowers or potential contact in large nest aggregations.

Drifting is a term used for bees with faulty orientation and homing, when bees enter a non-natal colony from the same species. This phenomenon is well described for honey bees (Pfeiffer and Crailsheim, 1998), but it also occurs in other bee species, e.g., *Halictus ligatus* (Packer, 1986), *Melipona scutellaris* (Alves et al., 2009) and *Bombus* sp. (Lopez-Vaamonde et al., 2004). Drifting exposes the intruding individual to the nest structures or pollen provisions of other individuals from the same species and may thus facilitate viral transmission.

#### Vector-Mediated Transmission

Another possible transmission route between non-*Apis* bees are parasitoids, parasites and commensals living in, on or with the host bee or its nest structures. Neither *V. destructor*, the highly potent vector of honey bee viruses in *A. mellifera* (Traynor et al., 2020) nor similar virus-transmitting mites parasitizing *Apis* bees in Asia (Forsgren et al., 2009, etc. see above in section “Vector-Mediated Transmission” in: Transmission of Viruses in *Apis mellifera*) parasitize non-*Apis* bees. However, non-*Apis* bees are host to a plethora of other parasites, ranging from ecto-parasites to parasitoids, that could potentially vector viruses. DWV has been identified in several species of parasitoid phorid flies, i.e., *Megaselia scalaris* and *Apocephalus borealis*, with evidence of replicating virus in the larvae of *Megaselia scalaris* (Core et al., 2012; Menail et al., 2016). *A. borealis* is a known parasitoid of bumble bees, which was also detected in honey bees. *M. scalaris* on the other hand is a broad host parasitoid, known to affect different insect species (Robinson, 1975; Mongiardino et al., 2013) and has also been suggested to be a parasitoid of Melipona colonies (Macieira et al., 1983). Even though DWV has been identified in these phorid flies, further research is still needed to establish the true potential of these species as an intra-specific and potentially inter-specific vector of bee viruses. The geographic expansion of the invasive SHB to temperate climatic zones means that it can now also encounter various bumble bee species, whose colonies it is able to invade (Hoffmann et al., 2008). Not least, it has been recently shown that SHBs can also complete an entire life cycle in association with nests of solitary bees *Megachile rotundata* (Gonthier et al., 2019). Since SHB is a replicative host or biological vector for DWV (Eyer et al., 2009), such invasion may result in the transmission of viruses among bee species.

### Transmission of Viruses in Non-bee Insects

#### Hymenoptera

Many species of the *Vespidae* family prey on bees and/or share nectar resources with Apoidea, this exposes them to bee-associated viruses. Several of these viruses have been detected in wasps of the *Vespidae* family. DWV has been reported in several species of the *Vespula* genus such as *V. vulgaris, V. pensylvanica, V. crabro, V. velutina*, and other *Vespula* spp. (Singh et al., 2010; Evison et al., 2012; Levitt et al., 2013; Forzan et al., 2017; Mazzei et al., 2018). Furthermore, DWV has also been detected in other genera of the *Vespidae* family such as *Polistes* spp. and *Bembix* spp. (Singh et al., 2010; Santamaria et al., 2018) the latter genus is mostly known to hunt flies (Evans, 2002), but is also described as a predator of stingless bees (Evans and O’Neill, 2007). Besides DWV other viruses have also been detected in wasp species, such as IAPV (Yañez et al., 2012b), Moku virus (Garigliany et al., 2017) ALPV, KBV, and BQCV (Mazzei et al., 2019; Yang et al., 2019). An RNAseq analysis of different tissues of Asian hornets, *V. velutina*, recently identified 18 virus species and added ABPV, BeeMLV and DWV-C to the list of bee associated viruses found in wasp species (Dalmon et al., 2019). Furthermore, the same study showed the presence of DWV, BQCV, and ABPV in tissues other than the gut, which suggests that these viruses cause a real infection in *V. velutina*. Similarly, the detection of virus replication of DWV, IAPV, KBV, and BQCV strongly suggests these viruses truly infect wasp species (Yañez et al., 2012b; Forzan et al., 2017; Mazzei et al., 2018, 2019).

Interestingly, a study in Hawaii by Loope et al. (2019) showed that the presence of *V. destructor* in *A. mellifera* has a cascade effect on the DWV variants found in *Vespula pensylvanica*. The arrival of *V. destructor* reduced the DWV variant diversity in honey bees as well as *V. pensylvanica*. These results further underline that predation of bees by wasps is a potential route of virus transmission. Furthermore, Mordecai et al. (2016b) showed that Moku virus is predominantly present in *V. pensylvanica* but is also found in honey bees and varroa in the same locations. Their data suggest that *V. pensylvanica* is likely the primary host of Moku virus, based on high viral loads and full genome recovery of the virus in the wasp. However, as they found the virus also in honey bees and *V. destructor*, it is also transmitted from wasps to bees and subsequently to varroa, the most likely route here is transmission via the use of shared flowers (Mordecai et al., 2016b).

The Moku virus, also highlights that for several viruses found in honey bees, the honey bees themselves may not be the primary host, yet they acquire the virus from other species. This has also been shown by de Souza et al. (2019), who found that DWV-C was more dominant in *Melipona subnitida* compared to *A. mellifera* in Brazilian colonies. Although the honey bee is often the focal host of studies, the detection and identification of honey bees as a potent host species for a virus does not directly imply that the honey bee is the primary host for this virus. Further research is still needed to untangle the role of different host species for most viruses found in honey bees.

The ants *Camponotus vagus* and *Formica rufa* feed on honeydew but also honey bee cadavers. Both species, when collected near apiaries, have been shown to carry CBPV which they could have acquired either through feeding on dead infected bees, or by sharing the same source of honeydew (Celle et al., 2008). Larvae and adult *Lasius platythorax* ants collected from honey bee colonies have been shown to carry ABPV, DWV-A, and DWV-B (Schläppi et al., 2020). Replicative strands of the CBPV and ABPV genomes were detected in *C. vagus* and in *L. platythorax*, respectively, suggesting possible viral replication and true host status for these ant and wasp species. However, as with *V. destructor*, such results should be interpreted with caution, since the virus (and its replication intermediates) may have come with the bee cadaver, rather than from the wasp/ant host tissues. Similarly, replicative strands of the DWV genome were detected in *Myrmica rubra* (Schläppi et al., 2019), an invasive species of ant in North America (Groden et al., 2005), and in the Argentine ant *Linepithema humile* (Sébastien et al., 2015), also one of the most widespread and abundant invasive ant species (Holway et al., 2002). Specific strains of LSV were detected in *Messor* ant species, which were thought to be genuine infections rather than the result of inter-species transmission (Bigot et al., 2017).

#### Coleoptera

The SHB, *Aethina tumida*, is a scavenger/parasite of honey bee colonies that was native in Africa, but has recently become invasive on several continents. Adult beetles enter honey bee and bumble bee colonies where they mate and reproduce. SHB feeds on the pollen, honey and honey bee brood and is therefore exposed to any virus found in these hive products (Neumann and Elzen, 2004). Additionally, the SHB can exploit trophallaxis and be fed directly by bees. Furthermore, replicative strands of the DWV genome have been detected in *A. tumida* (Eyer et al., 2009), although these can be either acquired passively through feeding or indicate active virus replication in SHB. BQCV, DWV, IAPV, KBV, and SBV, have been detected in *A. tumida* as well (Levitt et al., 2013). Additionally, DWV has been detected in individuals of the *Coccinellidae* and *Tenebrionidae* families (Levitt et al., 2013). However, further research is still needed to confirm the host status of Coleoptera for these viruses.

#### Diptera

The adults of some species of hoverfly (*Syrphidae, Diptera*) are considered to be Batesian mimics of adult honey bees. They feed on flowers, effectively imitate honey bee behavior (Golding and Edmunds, 2000) and share many floral resources with bees as adults (Power and Stout, 2011). It has been shown that some species of the genus *Eristalis* carry BQCV, SBV, and DWV-B, most likely acquired through horizontal transmission (Bailes et al., 2018). Levitt et al. (2013) reported the presence of DWV in samples of the *Calliphoridae* and the *Muscidae* families of flies.

#### Other Insects

Levitt et al. (2013) collected a large number of insects in the proximity of apiaries and screened them for the presence of five bee-associated viruses. In addition to the detection of bee viruses in various species of Hymenoptera, Coleoptera, and Diptera (as mentioned above), BQCV, DWV, IAPV, KBV, and SBV were all also detected in species of *Blattodea*, Dermaptera, and Lepidoptera, while only DWV was detected in a specimen of Pentatomidae. None of these viruses were detected in specimens of Odonata or Orthoptera, although only very few specimens of these orders were analyzed.

#### Other Arthropods

Several bee-associated viruses, BQCV, DWV, IAPV, and SBV, were detected in Arachnids sampled near apiaries. As with other parasites, parasitoids and commensals, it is unclear if these viruses represent true infections (and thus host status) or were acquired passively through feeding on infected bees or contaminated material (Levitt et al., 2013).

## Part 2: Artificial Infections

This section deals with the deliberate, experimental infection of bees with controlled amounts of virus, using a variety of inoculation techniques, trying to mimic a natural transmission route. This requires a source of relatively pure virus and, ideally, uninfected experimental bees. The success of the infection procedure is tested either through molecular evidence of replication of the inoculated virus, or more commonly a significant quantitative increase in post-inoculation virus titer that can only be attributed to the inoculum. A number of negative control inoculations are therefore also required, to rule out alternative sources of infection or virus titer increase.

### Sources of Virus Inoculum

The primary requirement for controlled inoculation is a source of relatively pure virus. There are two main approaches to achieving this: through the *in vivo* (bees) or *in vitro* (cell cultures) propagation of natural virus isolates (de Miranda et al., 2013; Genersch et al., 2013), or through reverse genetics, where the entire virus genome is transcribed synthetically from plasmid clones or PCR products and introduced into bees as full-length infectious RNA (Benjeddou et al., 2002; de Miranda et al., 2013; Lamp et al., 2016; Ryabov et al., 2019). Most studies on artificial inoculation of bee viruses thus far have been conducted with virus material propagated *in vivo*, in honey bee pupae, and enriched and purified through differential centrifugation (de Miranda et al., 2013). Because multiple virus infections are common in the bee colonies and most bee viruses have similar physico-chemical properties, making it impossible to separate them by differential centrifugation, these studies normally involved semi-pure virus inocula containing varying amounts of contaminating viruses (Bailey and Ball, 1991; Carrillo-Tripp et al., 2015; Remnant et al., 2019; Thaduri et al., 2019). Covert virus infections (virus present at very low levels) in either the propagating pupae, for preparing inoculum, or in the experimental bees can easily be co-amplified and interfere with the virus under study (Carrillo-Tripp et al., 2015; Remnant et al., 2019). Moreover, crude bee preparations also contain host cellular material that can independently or in synergy with either the inoculated or resident background viruses to influence the virus infection dynamics. An alternative approach would be to synthesize the virus of interest *in vitro* (Lamp et al., 2016; Ryabov et al., 2019; Seitz et al., 2019; Jin et al., 2020; Yang et al., 2020) thereby ensuring the highest level of purity. Both cell cultures and reverse genetics also allow virus to be produced that is free of contaminants, while reverse genetics also has the option of introducing specific genetic changes to the virus genome (Lamp et al., 2016; Ryabov et al., 2019; Jin et al., 2020). The combination of reverse genetics and cell culture propagation is particularly powerful for obtaining large amounts of pure infectious virus particles. However, despite persistent attempts during the last several decades, it was not until recently that cell culture systems and infection methods were optimized for honey bee virus infection and propagation (Genersch et al., 2013; Carrillo-Tripp et al., 2015) and full-length infectious plasmid clones of several honey bee viruses were developed (Yang et al., 2013; Lamp et al., 2016; Ryabov et al., 2019; Seitz et al., 2019). Recently, a molecular clone of CBPV was shown to cause typical clinical symptoms mimicking naturally CBPV-infected honey bees (Seitz et al., 2019). Similarly, SBV and DWV clones have been synthetized to express the enhanced green fluorescent protein (EGFP; Jin et al., 2020; Ryabov et al., 2020). Besides creating a clone that produces typical symptoms, it adds the advantage of a reporter gene for protein expression studies (Jin et al., 2020). One valid criticism of the reverse genetics approach is that it usually involves a single pure genetic clone, while viruses exist naturally as quasispecies – a collection of interrelated major variants, point mutants, recombinants and defective genomes (Dolan et al., 2018). Experiments with pure single genome viruses therefore lack the functional and genetic complexity of natural virus isolates. The obvious solution to this is to create a diverse set of infectious cDNA clones representing the genetic diversity of the original population (e.g., Ryabov et al., 2019).

Multiple positive and negative controls for all the steps of the inoculation process, from the manipulation of the individuals, incubation conditions, mode of inoculation, etc., are required to ensure that the infection is due to the target virus in the inoculum, and not due to contaminants in either the inoculum or the recipient host. The quality and quantity of the virus in the original inoculum as well as in the inoculated bees can be evaluated by qualitative and quantitative real-time PCR and by sequencing. The methods for virus propagation and virus infectivity assays have been described in detail in the Beebook (de Miranda et al., 2013). Here, we focus on summarizing the results of virus infectivity assays conducted during the last years.

Artificial infection experiments in honey bees primarily involve two forms of inoculation: by direct injection of micro volumes of virus into the honey bee using a fine needle, mimicking the vectored transmission by varroa, or by feeding, mimicking the oral-fecal transmission route. Very occasionally topical application is used, mimicking transmission by contact. Inoculation by injection allows absolute control over the amount of virus each bee receives, but not necessarily of the subsequent progression of the infection. This is because injection directly by-passes the main physical and physiological antiviral defenses, leaving the infection subject to only molecular controls. This is in contrast to oral inoculation, which often requires much higher amounts of virus (between 10^6^ and 10^11^ particles, depending on the virus; Bailey and Gibbs, 1964; Bailey and Ball, 1991; de Miranda et al., 2013) to initiate infection, due to these natural physical and physiological barriers, but whose subsequent progression is much more measured and predictable. The precise conditions for artificial inoculation vary greatly among viruses. Some of them have been studied widely while for others this information is largely unknown.

#### Oral Inoculation and Injections

##### DWV

Oral inoculation of adult bees with DWV does not induce overt DWV infections, even when using large titers of the virus (10^8^ genome equivalents), and the virus was restricted to the abdominal organs (Möckel et al., 2011). Artificial oral inoculation of DWV seems to be rather ineffective (Iqbal and Mueller, 2007), much of which can be attributed to the extreme instability of DWV in isolation (de Miranda et al., 2013). However, when feeding 2 days-old larvae with 2 μl of serial dilutions of DWV, Khongphinitbunjong et al. (2015) observed significant higher viral titers in the resulting adults than in sucrose-fed controls, suggesting that both the developmental stage used for oral infection and the virus quantity were significant for establishing infection. Similar results were obtained by Thaduri et al. (2019) for oral inoculation of larvae with a single dose of freshly prepared crude DWV extracts containing 10^8^–10^9^ DWV genome equivalents, although the majority of this would have been unpackaged cytoplasmic RNA and only a fraction from virus particles. The results for adult bees were equivocal due to the high background levels of DWV in newly emerged adult bees (Thaduri et al., 2019). Artificially reared newly hatched larvae orally inoculated with high doses of DWV (about 10^10^ virus genome equivalents) established high levels of DWV infection (Ryabov et al., 2016). Sequential experimental oral infections with DWV and *Nosema ceranae* in 2 days old workers resulted in lower DWV loads when *N. ceranae* was inoculated before DWV, suggesting competitive interference between pathogens (Doublet et al., 2015).

Overt DWV infections could only be obtained through injecting the virus into young pupae (Möckel et al., 2011; Natsopoulou et al., 2017; Dubois et al., 2020; Yañez et al., 2020), even with as little as 80 virus particles, but never through feeding (Möckel et al., 2011). Injection has been used frequently to mimic natural inoculation by varroa while feeding on nymphs or adult bees. Worker pupae at the white eye stage (12–13 days old) micro-injected with10^6^ copies of DWV exhibited virus replication and significant immune-gene expression modulations 5 days post injection (Ryabov et al., 2016), and injection of 10^7^ copies of either clone-derived DWV isolates or wild DWV isolates reached about 10^10^ to 10^11^ genome copies per bee after only 24 h post-injection (Ryabov et al., 2019). Similar results were obtained by Yañez et al. (2020) when serially injecting DWV into pink-eye pupae. When young adults were injected with 10^4^ to 10^6^ copies of DWV into the thorax or abdomen no acute mortality was observed but the bees’ lifespan decreased and flight behavior was affected (Mazzei et al., 2016; Bigot et al., 2017; Coulon et al., 2020). Both Natsopoulou et al. (2017) and Dubois et al. (2020) showed that DWV-A and DWV-B were equally capable of causing DWV symptoms, after injecting white-eye pupae and letting the pupae complete development *in vitro*. Mortality was significantly higher for adult honey bees injected with 10^7^ copies of DWV-B compared to bees injected with the same amount of DWV-A (McMahon et al., 2016). Gisder et al. (2018) showed that a DWV-B dominant isolate from mites changed genetic character to a DWV-A dominant derived isolate after a single passage in bee pupae, most likely through a simple quantitative shift in the relative levels of DWV-A and DWV-B genomes after passaging. The original DWV-B dominant isolate was more virulent than the evolved DWV-A dominant isolate when considering pupal mortality and adult bee cognitive behavior, but not for adult bee mortality. The elevated virulence of the DWV-B dominant isolate could be attributed to more efficient replication in pupae and wider dissemination in adult bee neurological tissues. The consensus sequences of matching source and passaged isolates with different virulence properties clustered differently with either DWV-A or DWV-B, depending on which region of the genome was analyzed, which allowed the elevated virulence to be mapped to the DWV-B RNA-dependent RNA polymerase (RdRp) region of the DWV genome (Gisder et al., 2018). Such differential phylogenetic affiliation across the DWV genome is most easily explained by a significant presence of recombinant viruses in the quasispecies, enough to change the genetic character of the consensus sequence of the whole isolate (Dolan et al., 2018). Such DWV-A/DWV-B recombinants are readily generated naturally in mixed infections (Moore et al., 2011; Zioni et al., 2011; Ryabov et al., 2014, 2019; Cornman, 2017; Dalmon et al., 2017) and have been used to map other differential traits of DWV-A and DWV-B as well (Moore et al., 2011; Ryabov et al., 2014, 2019).

##### SBV

Bailey et al. (1981) preferred injection to pupae up to 24 h old rather than injection in adult bees when infecting bees with BQCV, SBV, and SBPV. Early reports indicate that SBV extracted from larval or adult tissues (by grinding them in a 4:1 mixture of water and carbon tetrachloride followed by clarifying the extracts by centrifugation) were infectious to *A. mellifera*. Mature adult worker bees were infected by injection; newly emerged adult workers and larvae were infected by feeding (Bailey, 1969). Extrapolation from Bailey’s data indicates that about 10^7^-10^8^ viral particles fed to larvae resulted in above 90% mortality (Bailey, 1969; Bailey and Fernando, 1972). Electron microscope counts of the of virus particles from extracts of the heads of drones 5 days after injection indicated that about 7 × 10^11^ particles per drone-head, at least 100 times the number in an infected adult worker bee head (Bailey, 1969). Liu et al. (2010) reported that second instar *A. cerana* larva were fed with 5 μl SBV (Chinese SBV) solution or PBS (7.8 × 10^5^ genome copies/μl). Over 90% larval mortality was achieved by 96 h post infection. When comparing transmission for both DWV and SBV, Ryabov et al. (2016) used an inoculum of a mixture of approximately 10^10^ SBV and 10^10^ DWV genome equivalents for young larvae (oral infection), and 10^6^ SBV and 10^6^ DWV genome equivalents for pupae (injection).

##### BQCV

Oral inoculation was successfully used to inoculate pre-pupa (7 days) or 2 days old workers (respectively, 1.4 × 10^7^ BQCV genome equivalents per larva and 1.4 × 10^9^ genome equivalents per bee) to look for interactions between pathogens (BQCV and *N. ceranae*) and between the virus and pesticides (Doublet et al., 2015). Dose-dependent inoculation assays showed that only the high titers of BQCV (1.4 × 10^9^) caused higher mortalities of the larvae but no impact on adult worker survival was observed (Doublet et al., 2015).

##### ABPV, IAPV, KBV

Bailey et al. (1963) challenged adult honey bees with ABPV in three different ways: by feeding, spraying, and artificial injection of an ABPV suspension. The LD_50_ (number of ABPV particles per bee that would have killed half of the bees in a group after 6 days) was equivalent to about 10^2^ virus particles per bee when preparations were injected into the haemocoel compared to more than 10^11^ virus particles per bee for feeding and 10^8^ to 10^9^ particles per bee for spraying, illustrating the high virulence of ABPV upon injection.

Boncristiani et al. (2013) described that *in vitro* injection of honey bee pupae with 10^4^ viral genome equivalents of an IAPV-enriched extract resulted in symptomatic infection.

Oral infections with IAPV can be achieved by feeding IAPV-sucrose solution to honey bees in cage experiments and in honey bee colonies (Maori et al., 2007, 2009; Singh et al., 2010). Approximately 5–7 × 10^9^ IAPV genome equivalents in 30 ml sucrose were used to obtain colony infections) (Singh et al., 2010). IAPV-enriched sucrose solutions caused high mortality of recently emerged adult bees (concentrations above 10^8^ genomic copies per microliter, NC, unpublished).

An inoculum containing a mixture of viruses, enriched for SBV and IAPV with low levels of BQCV and DWV was able to produce high levels of mortality within 3 days of oral infection in newly emerged honey bees. Further investigation concluded that the bees died from the extremely high titers of IAPV, and not from other viruses (Carrillo-Tripp et al., 2016; Dolezal et al., 2016).

##### SBPV

Adult bees injected with preparations of SBPV and antisera to neutralize all the other known bee viruses died after about 12 days at 30 or 35°C. They suffer a paralysis of the legs a few days before death and contained about 10^12^ viral particles (Bailey and Woods, 1974).

##### CBPV

Blanchard et al. (2007) reported infection of adult bees after topical application of 1.8 × 10^8^ CBPV genome equivalents on the thorax of the bees. Trembling and weakening symptoms were observed 7–8 days post-application and all bees died 8–9 days post-contact (Blanchard et al., 2007). Queens can be infected by topical application of CBPV or following their exposure to CBPV-infected adults. The disease’s symptoms include trembling of the legs, spread and disjunct wings and sometimes bloated abdomens (Amiri et al., 2014). Coulon et al. (2018) set up contact transmission experiments, mimicking the natural transmission route in the hive that avoids the stress of shaving and topical application. Tagged bees previously injected (4 days earlier) into the thorax with 4 × 10^4^ CBPV genome equivalents were reared in the same cage with 9-day-old bees. Many of the bees that received the inoculum died after 1–3 days, but 69% of the bees that were in contact with them were still alive after 10 days despite the high titers of CBPV (about 10^8^ CBPV genome equivalents, Coulon et al., 2018).

#### Infection by Mites

After 6 days the larvae that was fed with larval food provided by nurse bees, which was naturally infected with DWV, showed lower DWV titers (about 10^6^ genome equivalents/larvae) than larvae exposed to *V. destructor* (Ryabov et al., 2014). Pupae artificially infested with one mite resulted in higher prevalence of DWV in adults than adults from non-infested pupae and DWV titers reached up to 10^10^ genome equivalents per bee even if the distribution of DWV titers across bees were highly varied.

Santillán-Galicia et al. (2014) investigated the ability of *V. destructor* to transmit SBPV. *A. mellifera* and mites were fed on SBPV-infected pupae for either 5 or 10 days. Using Probit analysis, they estimated doses necessary to cause 50% (LD_50_) and 99% (LD_99_) mortality in bees was 362 and 2266 virus particles, respectively. A time course of SBPV replication in pupae showed that the virus was first detected 42 h after injection. No significant differences were found in the overall proportion of pupae that became infected when mites were introduced over a period of 5 or 10 days, irrespective of the season (July or September). The proportion of pupae infected with SBPV declined significantly with each mite transfer over time, with the majority of pupae becoming infected after the first few mite transfers. These experiments suggest that transmission of SBPV does occur during mite feeding. Furthermore, they conclude that because SBPV is highly virulent as it kills the bees before any dramatic increase in virus titer. This results explains why low SBPV titers exist in honey bee colonies even in the presence of *V. destructor* (Santillán-Galicia et al., 2014).

### Cross-Infection Between Bee Species

#### *Apis* Species

Only a few studies have been conducted on artificially cross-infection of bee viruses between different *Apis* species, mostly due to the lack of *in vitro* brood rearing techniques for *Apis* species other than *A*. *mellifera*. Kashmir bee virus was originally found in Eastern honey bees, *A. cerana* in Kashmir (Bailey and Woods, 1977) and later found in *A. mellifera* in Australia (Bailey et al., 1979). Experimental work has shown that the virus could multiply very effectively in *A. mellifera* pupae. Inoculation with 1 × 10^–8^ ng of purified virus was sufficient to cause infection in some pupae, and at doses of 1 × 10^–5^ ng of purified virus every inoculated individual became infected. Virus multiplication occurred in cytoplasmic membrane-bound vesicles and caused significant changes in the hemolymph osmolality of infected pupae (Dall, 1987). Bailey also found that infected *A. mellifera* adult bees died within 3 days after either injecting or rubbing KBV on their bodies (Bailey et al., 1981).

Chinese sacbrood virus (CSBV) originating from *A. cerana* could readily establish SBV infections in *A. mellifera* larvae and adults, through both natural and artificial infection (Gong et al., 2016; Sun et al., 2017). Although CSBV was shown to replicate in *A. mellifera* adult bees and larvae, no obvious signs of sacbrood disease was observed (Gong et al., 2016).

#### Bombus Species

Many studies have shown that viruses first identified in honey bees could also be detected in *Bombus* and other pollinator species (e.g., Singh et al., 2010; Fürst et al., 2014; Gusachenko et al., 2019; see Gisder and Genersch, 2017 for an extensive overview). Several studies also report the active replication of these viruses in *Bombus* spp., indicating that these non-*Apis* bee species are also true hosts for these viruses. However, merely establishing that a virus can replicate in a bee species says nothing about either the pathology or the intra-species transmission of the virus. This additional biological information is crucial for evaluating the ecological consequences of virus prevalence in non-*Apis* pollinators. Establishing the pathology of bee viruses in non-*Apis* pollinators is often difficult, since the contact history of wild-caught bees is not known. Moreover, wild specimens often have multiple infections with several viruses and/or other pathogens have not yet been developed. Artificial infections allow the study of a certain virus in a controlled environment. They also provide insight on the transmission ability of viruses between species. Artificial (cross-species) infections can be performed either by direct injection of the virus in the bee, or through oral administration. In the following sections, we provide a short overview on the application of different infection techniques to look at cross species infection potential.

#### Micro-Injection

The micro-injection procedure used in bumble bees is similar to that used in honey bees. Firstly, bees are anaesthetized by putting them in the freezer for 10–20 min, subsequently bees are injected using a micro-capillary in the soft tissue between the first pair of sternites (Niu et al., 2016). Niu et al. (2014) showed that injection with as low as 20 virus particles of IAPV originating from honey bees results in infection in *B. terrestris.* Similarly, Wang et al. (2017) showed that injection of 500 virus particles of IAPV from honey bees results in an acute infection inducing rapid mortality in *B. terrestris.* The injection of SBPV particles, on the other hand, has a much less lethal effect on *B. terrestris.* Niu et al. (2016) showed that injecting up to 200,000 virus particles resulted in a moderately increased mortality compared to the control. Interestingly, both IAPV and SBPV inocula originating from white eyed honey bee pupae, reach a similar amount of viral genome copies in the bumble bee, yet IAPV appears much more lethal compared to SBPV (Niu et al., 2016). While the presence of DWV is documented in field samples of several *Bombus* species, published experimental work is still scarce. Graystock et al. (2015) showed that injections of DWV in *B. terrestris* result in an infection and lead to 50% mortality 10 days after injection.

Other than bumble bees, *Osmia* sp. have been used to test cross-species infectivity of DWV through injection. Mazzei et al. (2014) injected *O. cornuta* with extracts DWV infected honey bees. The bees were injected into the thorax, and later replicating DWV could be detected in the abdomen of the infected bees but not in their heads.

#### Feeding

Feeding virus particles to bee species other than honey bees, more closely resembles the natural transmission process, as vectors injecting virus are not described for non-*Apis* bees. In nature cross-species infection most likely occur due to feeding on flowers contaminated with fecal matter containing virus particles (*vide supra* for transmission pathways).

Establishing an infection via feeding requires much more viral particles compared to injection, as not all administered virus is able to penetrate the gut tissue and enter the haemocoel. Meeus et al. (2014) reported successful oral infection of *B. terrestris* with KBV and IAPV using 1 × 10^7^ and 0.5 × 10^7^ virus particles, respectively. Similar orders of magnitude were reported for oral infection of *B. terrestris* with IAPV in other studies (Piot et al., 2015; Wang et al., 2019). Both feeding and injection of IAPV highly increase mortality of *B. terrestris*, yet injection is far more lethal compared to feeding (Wang et al., 2019). Similarly, feeding bumble bees with DWV particles is less efficient at triggering an infection compared to injection. *B. terrestris* fed with 1 × 10^9^ of DWV particles show a significant infection in less than half of the individuals (Fürst et al., 2014). However, compared to honey bees, bumble bees can be housed alone without an increased mortality, when they have access to *ad libitum* sugar water.

Schläppi et al. (2020) reported oral transmission of ABPV to the black garden ant *Lasius niger* when feeding on highly infected honey bee pupae (2 × 10^11^ virus particles), which led to symptoms at colony (fewer emerging workers) and individual level (impaired locomotion and movement speed).

### Serial Transmission of Viruses in Honey Bees

Historically, starting with the work of Louis Pasteur on rabies in the 19th century, serial passage of viruses in alternative hosts has been used for attenuating their virulence, in order to develop low-virulence virus strains for use as live vaccines. The principle is that after the serial passage of a virus to another host species, a loss of virulence is experienced when inoculated again into the original host as the virus becomes adapted to the alternative host and less to the original host. However, serial passaging has also been used to select strains with increased virulence (e.g., HIV-2 in baboons, Locher et al., 2003) and as an experimental technique to study genetic adaptation in virus populations resulting in new viral population-level traits, such as the adaptation to new hosts.

Serial passaging of viruses from honey bees has thus far largely involved *in vivo* serial passaging in pupae and adult bees. This is mostly because immortal honey bee cell lines, a logical pristine environment for pure virus propagation, have not been available until recently (see the section “Cell Line in Honey Bees”). Serial passaging of bee viruses was initially used to identify the infectious agent associated with different disease symptoms in honey bees, most commonly paralysis, and subsequently also to maintain infective virus stocks for experimentation and raising diagnostic antisera. This work started in 1945 with single passages of extracts from paralytic bees (Burnside, 1945) and was developed more thoroughly during the 1960s–1970s, leading to the discovery and initial characterization of the most common and important bee viruses we know today (Bailey and Ball, 1991). For instance, in Bailey et al. (1963), stocks of ABPV and CBPV were maintained by serially injecting those viruses in adult bees. In the case of ABPV, the virus infectivity has been maintained by serial transmission for over two years. While CBPV has been maintained in a similar way for several months.

As mentioned above, serial passaging can be used to either decrease or increase virulence. Mussen and Furgala (1977) compared the virulence of several SBV extracts, including one obtained from symptomatic larvae and another from SBV serially transmitted through adult bees. Both extracts were then inoculated by injection into 1-day-old adult bees, with higher mortality observed with the extracts from adult bees (100% mortality at 14 days after inoculation for adult-derived SBV, compared to 75% from symptomatic larvae). Similar experiments have recently also been conducted with the two master variants of DWV, comparing the relative virulence in honey bee pupae of DWV-A, the original honey bee specific strain (Lanzi et al., 2006; Posada-Florez et al., 2019), and DWV-B, a strain adapted to and capable of replicating in the parasitic mite and virus vector *V. destructor* (Ongus et al., 2004; Ryabov et al., 2019). In one study, no differences were observed in virus titers, development of symptoms or mortality between bees infected by DWV-A and DWV-B separately or infected by the mixture of both, through a single passage in white-eyed pupae (Tehel et al., 2019). In a different study, a single passage in pupae of a DWV isolate from a crippled bee significantly attenuated its virulence when injected into pupae (mortality) and in adult bees (neurotropism and cognitive ability), with attendant genetic changes in the virus linking higher virulence to sequence signatures from the DWV-B genotype, particularly the RNA-dependent RNA polymerase region (Gisder et al., 2018). As discussed above, this change in virulence could also be due to the presence of recombinants in a mixed infection. Virulence attenuation has also been demonstrated for varroa-mediated serial transmission, with reduced pupal DWV-A titers in pupae after just three varroa-mediated passages (Posada-Florez et al., 2019). These experiments demonstrate that artificial injection is useful for the transmission and propagation of viruses but may not reflect the natural interactions between each component within the three-way interactions between DWV, varroa and honey bees.

Serial passages were also used to evaluate viral species interaction during co-infection of the same host (Ryabov et al., 2016; Remnant et al., 2019). Virus isolates propagated *in vivo* in natural pupae inevitably include other common co-purifying viruses, such as SBV and BQCV, especially when propagating DWV (de Miranda et al., 2013) due to its innate instability in isolation (Lanzi et al., 2006). This will naturally also affect the replication and virulence characteristics associated with the propagated inoculum. From the first passages onward, DWV-A was outcompeted by SBV and BQCV (Remnant et al., 2019) both of which replicate very efficiently upon injection (Bailey and Ball, 1991). These unique virus-specific differences revealed by competition between co-replicating viruses are also reflected in both common and virus-specific host molecular responses to co-infection with competing viruses (Ryabov et al., 2016).

Serial transmissions have also been used to test different transmission routes. For instance, Bailey and Gibbs (1964) tested the infectivity of ABPV through serial passages of bee feces. A hundred bees were each fed with 10^6^ ABPV particles. After 1 week, fifty bees were fed with syrup containing feces from the first group. The subsequent week, a serial transmission continued using another fifty bees fed with the feces from the second bee group. Those third group bees showed no symptoms of ABPV, nor was there any increase of virus or sign of disease during two further serial transfers. From these results, the author speculates that “when bees ingest feces while cleaning the hive, they become infected, but will be unlikely to receive enough virus to become acutely paralyzed.”

Serial passages have been also used to elucidate the behavior of viral quasispecies during and after transmission. Yañez et al. (2020) followed the changes in the DWV-A quasispecies shape upon serial injection into honey bee pupae. The results suggested that DWV-A quasispecies undergoes a rapid, extensive and random expansion of its sequence space, followed by very strong negative selection toward a uniform, common shape by the time the pupae have completed their development, with no particular signature between symptomatic and asymptomatic adults.

Serial passages of viruses have been a useful tool to preserve the viability of virus and to understand the different routes of transmission during the early virology research on the honey bees. In recent years, a few studies used this technique to characterize the complex interactions between co-infecting viruses, *V. destructor* and the honey bee host.

### Cell Line in Honey Bees

Following the initial attempts of creating *in vitro* insect cell cultures, the first primary cell line of continuously dividing insect cells was achieved in the 1960s (Grace, 1962), and since then, the field has grown to routinely cultivate primary cells and now immortalized or permanent insect cell lines (Lynn, 1999; van Oers and Lynn, 2010). According to the ExPASY Cellosaurus, thus far, around 1000 insect-derived cell lines have been established from several different tissue sources of many insect species, majority derived from the families Lepidopteran, Dipteran and Hemipteran (Lynn, 1999). Although there has been less success in the family Hymenoptera (wasps, ants, and bees) (Lynn, 2001), recent attempts have led to the first honey bee immortal cell line, AmE-711 (Goblirsch et al., 2013).

Honey bee primary cell cultures grow relatively slowly compared to other insect or animal cell cultures, regardless of the tissues used to initiate the cell culture (Genersch et al., 2013). Thus far, many attempts have resulted in several honey bee cell culture methods. These methods are highly varied as they use different target tissues, growth media and isolation methods (Bergem et al., 2006; Hunter, 2010; Ju and Ghil, 2015). Honey bee primary cell cultures have been established using different life stages from egg to adult bees and various isolated tissues including neural cells, antennae, fat body, hemocyte, and embryos (Kreissl and Bicker, 1992; Gascuel et al., 1994; Goldberg et al., 1999; Sorescu et al., 2003; Barbara et al., 2008; Ju and Ghil, 2015). Even transfection using human c-myc proto-oncogene into embryonic honey bee cells has generated a cell culture that remained viable for periods up to 8 months (Kitagishi et al., 2011). This cell line was considered as “of honey bee character” despite the expression of a central transcription factor being of human origin which is known to change the entire cellular program by un-regulating the expression of many genes (Nasi et al., 2001; Adhikary and Eilers, 2005). A major breakthrough in the development of a stable honey bee cell line came from the use of honey bee embryonic tissue (Goblirsch et al., 2013; Ju and Ghil, 2015), however, this and likely other primary honey bee cell cultures are plagued with persistent DWV infection, a condition afflicting honey bees worldwide (Martin and Brettell, 2019).

Insect-derived cell cultures have advanced our understanding of insect physiology, development biology, pathology, and molecular biology (Lynn, 1999; van Oers and Lynn, 2010). With their genetic uniformity, they are now used by scientists as a convenient tool to eliminate environmental variables with more consistent results that are impossible to control when working at the organismal or colony levels (Hunter et al., 2003; van Oers and Lynn, 2010). Cell cultures are desirable for detection, identification and isolation of many viruses and intracellular parasites in animals (Leland and Ginocchio, 2007; Hematian et al., 2016). They are especially valuable for rapid characterization of virus–virus, virus–host cellular interactions and their impact on cell survival, which could be commercially important (Carrillo-Tripp et al., 2015). That said, isolation of viruses from naturally infected bees and pupae, or artificially infected individuals in the laboratory (de Miranda et al., 2013), is still the preferred route for virus characterization studies because it is well known that immortalized cell cultures don’t reflect the true evolutionary pressure presented to a virus cultivated *in situ* or *in vivo*. Thus far, honey bee virus studies have provided valuable information on the response of hosts to viruses at the population and physiological levels but immortalized driven honey bee cell lines will provide a stable supply of material to a nascent field of honey bee cellular virology (de Miranda et al., 2013; Genersch et al., 2013; Martin and Brettell, 2019). It will also lead to better understanding of honey bee antiviral defense mechanisms (Carrillo-Tripp et al., 2016). Recently, primary cell cultures derived from of both Asian and European honey bees embryonic tissues were used to investigated cellular responses to virus infection (Goblirsch et al., 2013; Xia et al., 2014; Ju and Ghil, 2015). A monolayer of the primary cell culture from embryonic tissues of *A. cerana* was used to study early infection process of CSBV during replication (Xia et al., 2014). The results from this study suggest that after viral adsorption and entry into the host cell, CSBV replicated and assembled progeny virions in the cytoplasm until 48 hpi, after which CSBV particles might be released from the host cell by lysis. An investigation of viral co-infection in the immortal cell culture from embryonic tissues of *A. mellifera* (AmE-711) revealed a similar virus dynamic as individual honey bee (Carrillo-Tripp et al., 2016). These results indicated that different mechanisms of virus-host interaction affect virus infection dynamics, including virus–virus interactions, superinfections, specific virus saturation limits in cells and virus specialization for different cell types (Carrillo-Tripp et al., 2016).

## Conclusion

Although much is known about both the natural transmission routes and the artificial propagation of bee and bee-related viruses, much more still needs to be clarified. Most of our knowledge about bee virus transmission and infection has been derived from the most common honey bee viruses, i.e., those that also cause disease. The challenge is to elucidate the infection and transmission strategies of the much larger number of apparently non-pathogenic newly discovered viruses, as well as their biological and ecological importance to their hosts. Many of them will probably also use the most common transmission routes identified for the disease-causing viruses. The developmental stage and tissues targeted by these viruses will be important for both their transmission strategy and their effects on the host. Mechanical and biological virus vectors can breach the anatomical and physiological host barriers to virus transmission, with potentially drastic consequences for host health, virus virulence evolution, and applied vector-virus virulence management (Traynor et al., 2020). Also important in virus transmission, as recently reported by Wang et al. (2020) using a metabolomics-based approach, is the occurrence of diametrically opposite changes during virus infection of cells of different species origin, and we believe this phenomenon is possibly related to the type of infection (acute or persistent) that is triggered by the virus. Indeed Santos et al. (2019) and Wang et al. (2019) reported that virions/virus in insect species of three different orders (Lepidoptera, Hymenoptera, and Orthoptera) did not trigger pathogenic infections, while in Dipteran cells there were strong toxic effects (Wang et al., 2020), which can impact transmission among species in nature when sharing food resources. Indeed, Piot et al. (2019) demonstrated the impact of food hot spots on pathogens transmission within altered flower-networks that could negatively impact hosts experiencing an increased exposure. Finally, techniques revised here as inoculation methods, virus serial passaging and cell culture are all important tools for understanding virus quasispecies behavior, transmission, pathogenicity and virulence or its attenuation in different host bee species.

## Author Contributions

OY, AD, and NC conceptualized the study. OY, NP, AD, JM, DP, and NC investigated the study. OY, NP, AD, and NC contributed to methodology. OY, NP, AD, JM, PC, DP, EA, GS, DS, and NC wrote the original draft. OY, NP, AD, JM, PC, DP, EA, GS, and NC reviewed and edited the manuscript. OY, NP, AD, JM, PC, DP, EA, GS, DS, and NC approved the final version of manuscript to be published.

## Conflict of Interest

The authors declare that the research was conducted in the absence of any commercial or financial relationships that could be construed as a potential conflict of interest.
